# Editor's choice

**Published:** 2021-05

**Authors:** James K Tumwine

Welcome to this Afya Bora supplement of *African Health Sciences*. The supplement epitomizes successful completion of training of African fellows in Global leadership. The fellows have authored diverse manuscripts in three themes: HIV, reproductive and child health.

This is a welcome break from the COVID-19 pandemic. The HIV papers are basically on antiretroviral therapy (ART), with special emphasis on outcome^1^, quality of life^2^, adherence^3^,^4^, predictors of ART interruption^5^, and retention in care^6^. These are followed by papers on safe births^7^ and emergency obstetric acre^8^. The treatise ends with papers on the uptake of infant male circumcision^9^ and the WHO TB screening tool for children.^10^ Clearly this is an important milestone for the fellows and their mentors, all of whom deserve our congratulations.
